# A novel defined pyroptosis-related gene signature predicts prognosis and correlates with the tumour immune microenvironment in lung adenocarcinoma

**DOI:** 10.1038/s41598-023-36720-1

**Published:** 2023-06-19

**Authors:** Zi Chen, Linyang Ge, Shuanglan Xu, Qin Li, Linfu Zhou

**Affiliations:** 1 Department of Respiratory and Critical Care Medicine, The First Affiliated Hospital, Nanjing Medical University, Nanjing, Jiangsu China; 2 Guangzhou Municipal Research Institute of Clinical Medicine, Guangzhou First People’s Hospital, South China University of Technology, Guangzhou, Guangdong China; 3 Center of Molecular and Cellular Oncology, Yale Cancer Center, Yale School of Medicine, New Haven, CT USA; 4 Institute of Integrative Medicine, Nanjing Medical University, Nanjing, Jiangsu China

**Keywords:** Cancer, Lung cancer, Non-small-cell lung cancer, Cell death

## Abstract

Lung adenocarcinoma (LUAD) is one of the most common causes of cancer-related death. The role of pyroptosis in LUAD remains unclear. Our study aimed to identify a prognostic signature of pyroptosis-related genes (PRGs) and explore the connection of PRGs with the tumour microenvironment in LUAD. Gene expression and clinical information were obtained from The Cancer Genome Atlas database. Consensus clustering was applied to classify LUAD patients. The least absolute shrinkage and selection operator Cox and multivariate Cox regression models were used to generate a PRG-related prognostic signature. The correlations between PRGs and tumour-infiltrating immune cells or the tumour mutational burden were analysed by Spearman’s correlation analysis. In this study, 44 PRGs significantly differed in expression between LUAD and normal tissues. Based on these genes, patients were clustered into three clusters with significantly different distributions of tumour-infiltrating immune cells and immune checkpoint regulators. A total of four PRGs (*NLRP1*, *HMGB1*, *CYCS*, and *BAK1*) were used to construct a prognostic model. Significant correlations were observed between these prognostic PRGs and immune cell infiltration or the tumour mutational burden. Predictive nomogram results showed that *BAK1* could be an independent prognostic biomarker in LUAD. Additionally, the expression level of *BAK1* was validated in two independent Gene Expression Omnibus cohorts. Our identified prognostic PRG signature may provide insight for future studies targeting pyroptosis and the tumour microenvironment in LUAD. Future studies are needed to verify our current findings.

## Introduction

Lung cancer is one of the leading causes of death in the world, with an estimated 2.09 million new cases and 1.76 million deaths each year^1,2^. Non-small cell lung cancer (NSCLC) is one of the most common types, accounting for approximately 80% of all lung cancer cases, and can be further classified into three subtypes: lung squamous cell carcinoma (LUSC), lung adenocarcinoma (LUAD) and large-cell carcinoma^2–4^. LUAD is the most common histologic subtype, with an extremely poor survival rate ranging from 4 to 17%^5^. Although substantial progress has been made in the diagnosis and treatment of LUAD, the 5-year survival rate has been slow to improve^5,6^. Therefore, developing a novel and efficient prognostic model is important for the treatment of LUAD.

Pyroptosis is a newly identified type of programmed cell death characterized by rapid rupture of the cellular membrane and release of proinflammatory intracellular contents; it is thus also known as cellular inflammatory necrosis^7^. During pyroptosis, the inflammasome, caspase and gasdermin families are key executors^7–9^. The process comprises canonical and noncanonical regulatory pathways. The canonical pathway is initiated by activation of caspase-1 either directly or through recruitment of apoptosis-related speck-like proteins (ASC), followed by cleavage of gasdermin D (GSDMD) and further exposure of the N-terminal domain with pore formation, resulting in the release of intracellular contents, especially IL-1β and IL-18. In the noncanonical pathway, CASP-11 (CASP-4 or CASP-5 in humans) can be activated directly by cytosolic LPS from gram-negative bacteria, resulting in cleavage of GSDMD to induce cell pyroptosis^10–12^.

The relationship between pyroptosis and cancer is complex since the release of inflammatory mediators can form a microenvironment suitable for tumour cell growth, while on the other hand, as a type of cell death, the induction of tumour pyroptosis can inhibit the occurrence and development of tumours^9,12–14^. Therefore, pyroptosis is thought to play a dual role in tumours. In NSCLC, a higher expression level of GSDMD is related to invasive features, including an advanced tumour-node-metastasis stage and enlarged tumour size^12,15^. Downregulation of gasdermin A (GSDMA) results in caspase-3 activation and cancer cell death through the mitochondrial apoptotic pathway^16,17^. Researchers also found that GFNA5/GSDMD could determine the caspase-3-activated cell death mode and drug reactivity^12,18^. Some studies have explored the roles of pyroptosis-related genes (PRGs) in LUAD. Song et al*.*^19^ constructed a pyroptosis-related lncRNA signature to predict prognosis in LUAD patients. Lin et al*.*^20^ and Liu et al*.*^21^ each applied the same pyroptotic gene set consisting of 33 PRGs to investigate the roles of these genes in LUAD. These studies provide convincing evidence that pyroptosis is closely connected with LUAD pathogenesis.

In this study, based on an improved pyroptosis-related gene set consisting of 52 genes, we clustered LUAD patients from The Cancer Genome Atlas (TCGA) database and explored the tumour immune status within each subclass. Then, we developed a prognostic signature using the least absolute shrinkage and selection operator (LASSO) Cox method and studied the correlations between prognostic PRGs and the tumour mutational burden (TMB) or tumour-infiltrating immune cells. The expression level of *BAK1*, a potential independent predictor in LUAD, was validated in two independent Gene Expression Omnibus (GEO) datasets. Our findings indicate the potential connections of pyroptosis with disease prognosis and the immune microenvironment in LUAD patients.

## Materials and methods

### Acquisition of gene expression and clinical data

We obtained the RNA-sequencing data of 513 LUAD patients and corresponding clinical information from TCGA database (https://portal.gdc.cancer.gov/repository). Data and figures were analyzed and generated by R software (version v4.0.3; https://www.r-project.org/).

### Identification of differentially expressed PRGs

A total of 52 PRGs were obtained from previous reviews^10,15,22–26^, as shown in Table 1. We included 49 normal and 513 tumour tissue samples to identify PRGs. The ‘limma’ package was used to identify PRGs with an adjusted *p*-value < 0.05 and |log2FC|≥ 1. The PRGs are noted as follows: * if *p* < 0.05, ** if *p* < 0.01, and *** if *p* < 0.001.

**Table 1:** Full names of pyroptosis-related genes.

Genes	Full-names
*BAK1*	BCL2 antagonist/killer 1
*BAX*	BCL2 associated X, apoptosis regulator
*CASP1*	Caspase 1
*CASP3*	Caspase 3
*CASP4*	Caspase 4
*CASP5*	Caspase 5
*CHMP2A*	Charged multivesicular body protein 2A
*CHMP2B*	Charged multivesicular body protein 2B
*CHMP3*	Charged multivesicular body protein 3
*CHMP4A*	Charged multivesicular body protein 4A
*CHMP4B*	Charged multivesicular body protein 4B
*CHMP4C*	Charged multivesicular body protein 4C
*CHMP6*	Charged multivesicular body protein 6
*CHMP7*	charged multivesicular body protein 7
*CYCS*	Cytochrome c, somatic
*ELANE*	Elastase, neutrophil expressed
*GSDMD*	Gasdermin D
*GSDME*	Gasdermin E
*GZMB*	Granzyme B
*HMGB1*	High mobility group box 1
*IL18*	Interleukin 18
*IL1A*	Interleukin 1 alpha
*IL1B*	Interleukin 1 beta
*IRF1*	Interferon regulatory factor 1
*IRF2*	Interferon regulatory factor 2
*TP53*	Tumour protein p53
*TP63*	Tumour protein p63
*AIM2*	Absent in melanoma 2
*CASP6*	Caspase 6
*CASP8*	caspase 8
*CASP9*	Caspase 9
*GPX4*	Glutathione peroxidase 4
*GSDMA*	Gasdermin A
*GSDMB*	Gasdermin B
*GSDMC*	Gasdermin C
*IL6*	Interleukin 6
*NLRC4*	NLR family CARD domain containing 4
*NLRP1*	NLR family pyrin domain containing 1
*NLRP2*	NLR family pyrin domain containing 2
*NLRP3*	NLR family pyrin domain containing 3
*NLRP6*	NLR family pyrin domain containing 6
*NLRP7*	NLR family pyrin domain containing 7
*NOD1*	Nucleotide binding oligomerization domain containing 1
*NOD2*	Nucleotide binding oligomerization domain containing 2
*PJVK*	Pejvakin
*PLCG1*	Phospholipase C gamma 1
*PRKACA*	Protein kinase cAMP-activated catalytic subunit alpha
*PYCARD*	PYD and CARD domain containing
*SCAF11*	SR-related CTD associated factor 11
*TIRAP*	TIR domain containing adaptor protein
*TNF*	Tumour necrosis factor
*GZMA*	Granzyme A

### Estimation of immune cell type enrichment

Immune cell enrichment analysis of the RNA-seq data was performed with xCell. Relative cell type abundance was quantified and visualized across all samples. The abundance of each cell type across the clusters was compared using the Kruskal‒Wallis test. Cell types with *p* < 0.05 were considered significantly differentially enriched.

### Development of a PRG-based prognostic model

Cox regression analysis was performed to evaluate the prognostic roles of PRGs. Kaplan‒Meier survival analysis was applied to compare survival difference between two groups, with the *p*-values and hazard ratios (HRs) of 95% confidence intervals (CIs) generated by log-rank tests and univariate Cox proportional hazards regression. PRGs with significant prognostic values were included in further analyses. LASSO Cox regression analysis was used to construct a prognostic model based on the identified PRGs. The LUAD patients were divided into low- and high-risk clusters according to the median risk score, and the overall survival (OS) time was compared by Kaplan‒Meier analysis. Predictive accuracy was evaluated by performing time receiver operating characteristic (ROC) curve analysis.

### Construction of a gene-based prognostic nomogram

A composite nomogram was constructed based on the results of multivariate Cox proportional hazards analysis to predict 1-, 3-, and 5-year overall recurrence. A forest plot was used to present the *p*-value, HR, and 95% CI of each variable via the ‘forestplot’ R package.

### Immune infiltration and tumour mutational burden (TMB) analysis

The correlation between each prognostic PRG and tumour-infiltrating immune cells was analysed by Tumour Immune Estimation Resource (TIMER, https://cistrome.shinyapps.io/timer/), a web portal for comprehensive analysis of tumour-infiltrating immune cells. Spearman’s correlation analysis was applied to describe the correlation between each prognostic PRG and the TMB. A *p*-value less than 0.05 was considered statistically significant.

## Results

### Identification of differentially expressed PRGs between normal and LUAD tissues

**Figure 1:**
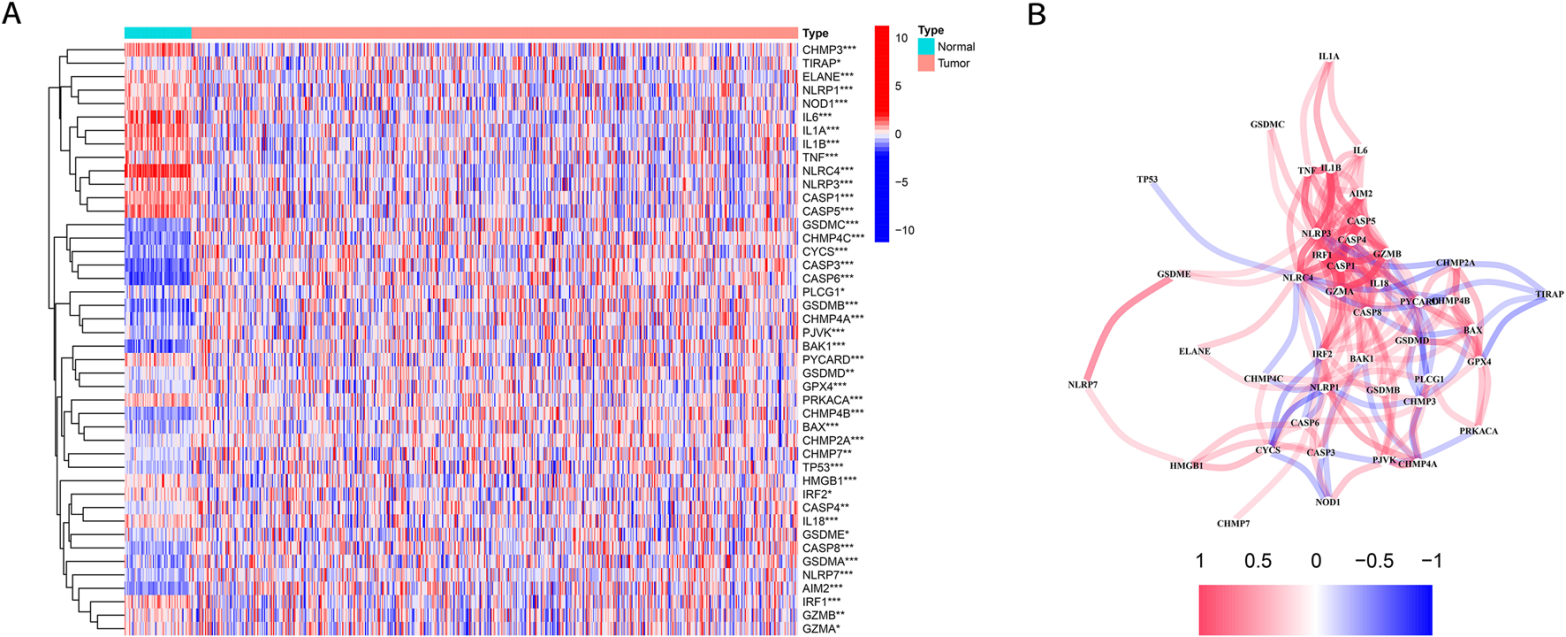
Expression of the 44 PRGs and the interactions among them. (**A**). Heatmap (red: high expression level; blue: low expression level) of the PRGs between normal (brilliant blue) and tumour tissues (red). *p*-values were shown as: ** *p* < 0.01; *** *p* < 0.001. (**B**). The correlation network of the PRGs (red line: positive correlation; blue line: negative correlation). The depth of the colours reflects the strength of the relevance.

The expression levels of 53 PRGs were compared between 49 normal and 513 LUAD tissues from the TCGA. Forty-four PRGs were significantly differentially expressed (Fig. 1A). The expression of *GSDMC*, *CHMP4C*, *CYCS*, *CASP3*, *CASP6*, *PLCG1*, *GSDMB*, *CHMP4A*, *PJVK*, *BAK1*, *PYCARD*, *GSDMD*, *GPX4*, *PRKACA*, *CHMP4B*, *BAX*, *CHMP2aA*, *CHMP7*, *TP53*, *HMGB1*, *IRF2*, *CASP4*, *IL18*, *GSDME*, *CASP8*, *GSDMA*, *NLRP7*, *AIM2*, *IRF1*, *GZMB*, and *GZMA* was increased in LUAD compared with normal tissues, while the expression of *CHMP3*, *TIRAP*, *ELANE*, *NLRP1*, *NOD1*, *IL1A*, *IL1B*, *TNF*, *NLRC4*, *NLRP3*, *CASP1*, and *CASP5* was decreased in LUAD compared with normal tissues. The correlation network containing all PRGs is presented in Fig. 1B.

### Identification of LUAD clusters by consensus clustering

**Figure 2:**
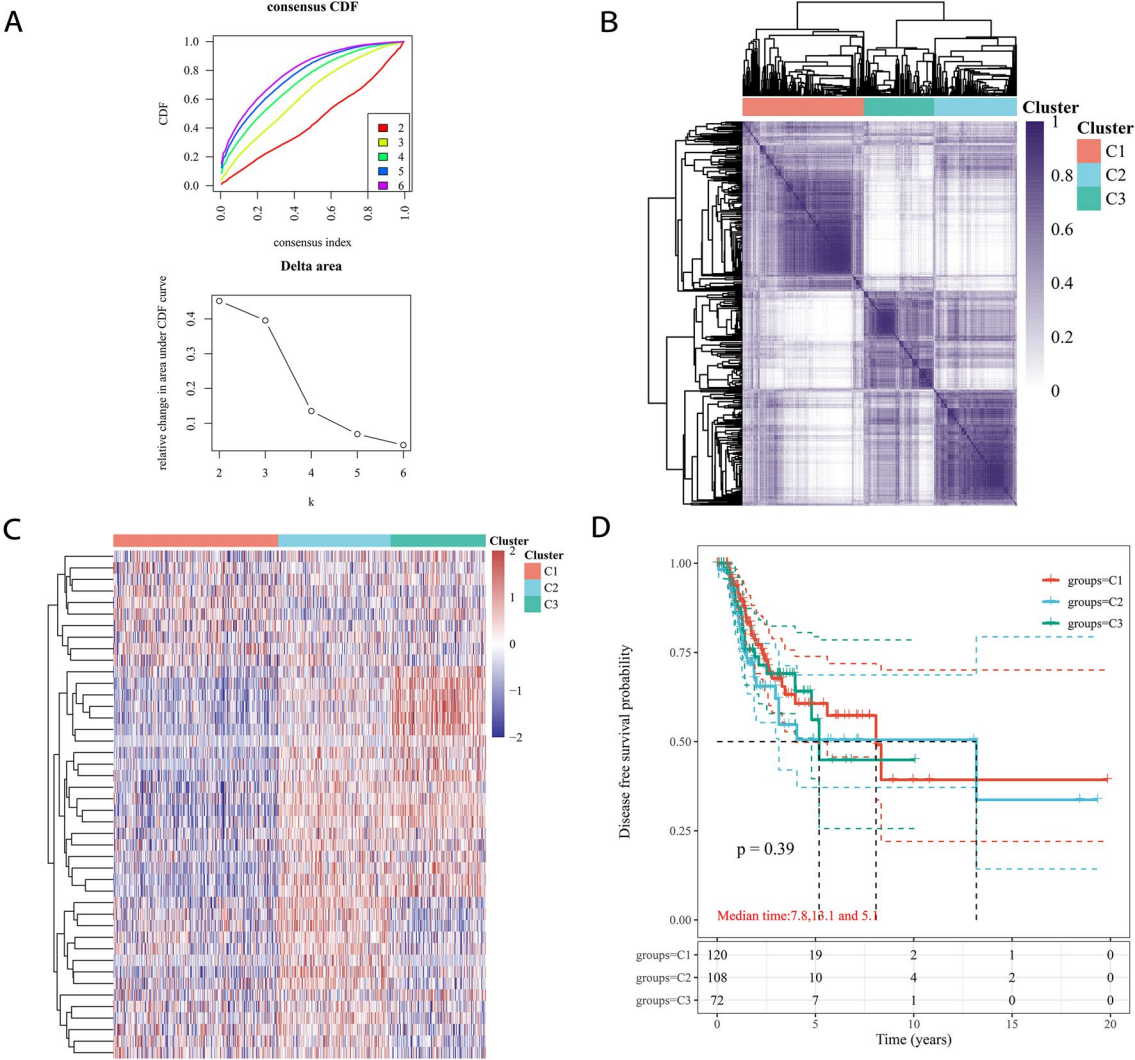
Tumour classification based on PRGs. (**A**, **B**). 513 LUAD patients were grouped into three clusters according to the consensus clustering matrix (k = 2). (**C**). Heatmap of the three clusters classified by these PRGs. (**D**). Kaplan–Meier OS curves for the three clusters.

To explore the connections between the 44 PRGs and LUAD subtypes, we performed consensus clustering analysis of all 513 LUAD patients in the TCGA cohort. The number of clusters was represented by the parameter ‘k’. The empirical cumulative distribution function (CDF) was plotted to determine the optimum k value for the sample distribution to reach maximal stability (Fig. 2A). By increasing k from 2 to 6, we found that when k = 2, LUAD patients could be divided into three distinct and nonoverlapping clusters (Fig. 2B). The differential gene expression profile is presented in a heatmap (Fig. 2C). The OS time was compared among the three clusters, and no obvious differences were found (*p* = 0.39, Fig. 2D).

### Distinct TMEs of PRG-based clusters

**Figure 3:**
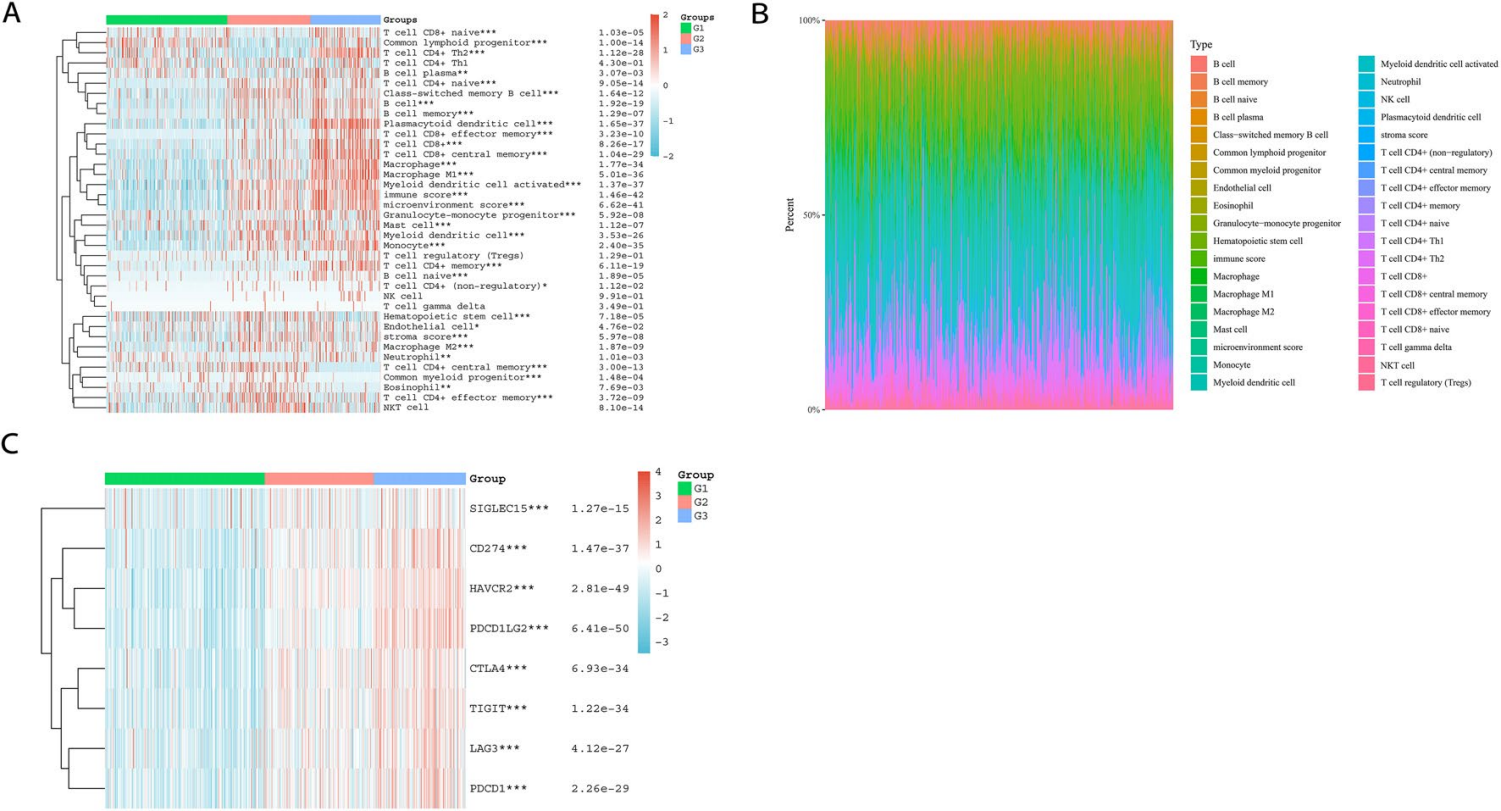
Tumour-infiltrating immune cells and immune checkpoint-related genes distribution in the three clusters. (**A**) Immune cell score heatmap with colours representing the expression trend in the three clusters. *p*-values were shown as: *p < 0.05; **p < 0.01; ***p < 0.001. (**B**) The percentage abundance of tumour-infiltrating immune cells in each sample. The abscissa represents the sample, and the ordinate represents the percentage of immune cell content in each sample. (**C**) The heatmap of immune checkpoint-related genes, where the horizontal axis represents each sample in the three clusters, and the vertical axis represents immune checkpoint-related gene expression. *p*-values were shown as: *p < 0.05; **p < 0.01; ***p < 0.001.

Next, we analysed tumour-infiltrating immune cell data and found that most of the infiltrating immune cells were abundant in Cluster 3, which included T cells (naive CD8^+^ T cells, effector memory CD8^+^ T cells, central memory CD8^+^ T cells, central memory CD4^+^ T cells, and effector memory CD4^+^ T cells), B cells (naive B cells, plasma B cells, class-switched memory B cells, and memory B cells), macrophages (M1 and M2), dendritic cells (activated myeloid dendritic cells and plasmacytoid dendritic cells), monocytes, mast cells, neutrophils and eosinophils (Fig. 3A). We also found that the proportions of immune cells within each of the three clusters were different, while the composition types were the same (Fig. 3B).

We also checked the expression of immune checkpoint-related genes (*SIGLEC15*, *CD274*, *HAVCR2*, *PDCD1LG2*, *CTLA4*, *TIGIT*, *LAG3*, and *PDCD1*) within the 3 clusters. The results revealed an expression profile similar to that of tumour-infiltrating immune cells in the 3 clusters. As shown in the immune checkpoint-related gene expression heatmap, where different colours represent the expression trends in each sample, the expression of immune checkpoint-related genes was most enriched in Cluster 3 (Fig. 3C).

### Construction of a PRG-based prognostic model

**Figure 4:**
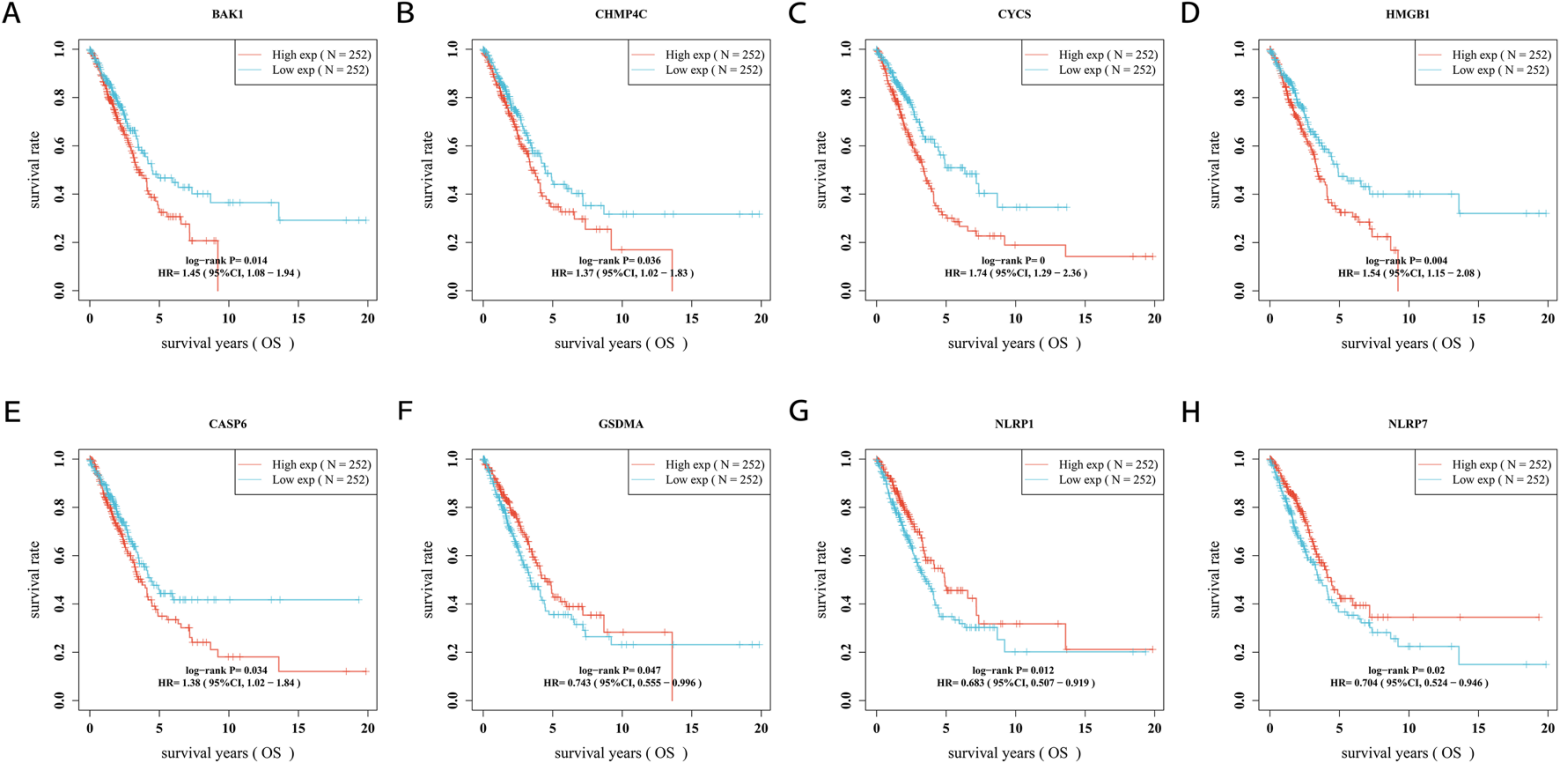
The Kaplan–Meier survival analysis for each prognostic PRG. The OS curve of *BAK1* (**A**), *CHMP4C* (**B**), *CYCS* (**C**), *HMGB1* (**D**), *CASP6* (**E**), *GSDMA* (**F**), *NLRP1* (**G**), and *NLRP7* (**H**) in LUSC patients in the high-/low-expression group. The *p*-values and hazard ratio (HR) with 95% confidence interval (CI) were generated by log-rank tests and univariate Cox proportional hazards regression.

**Figure 5:**
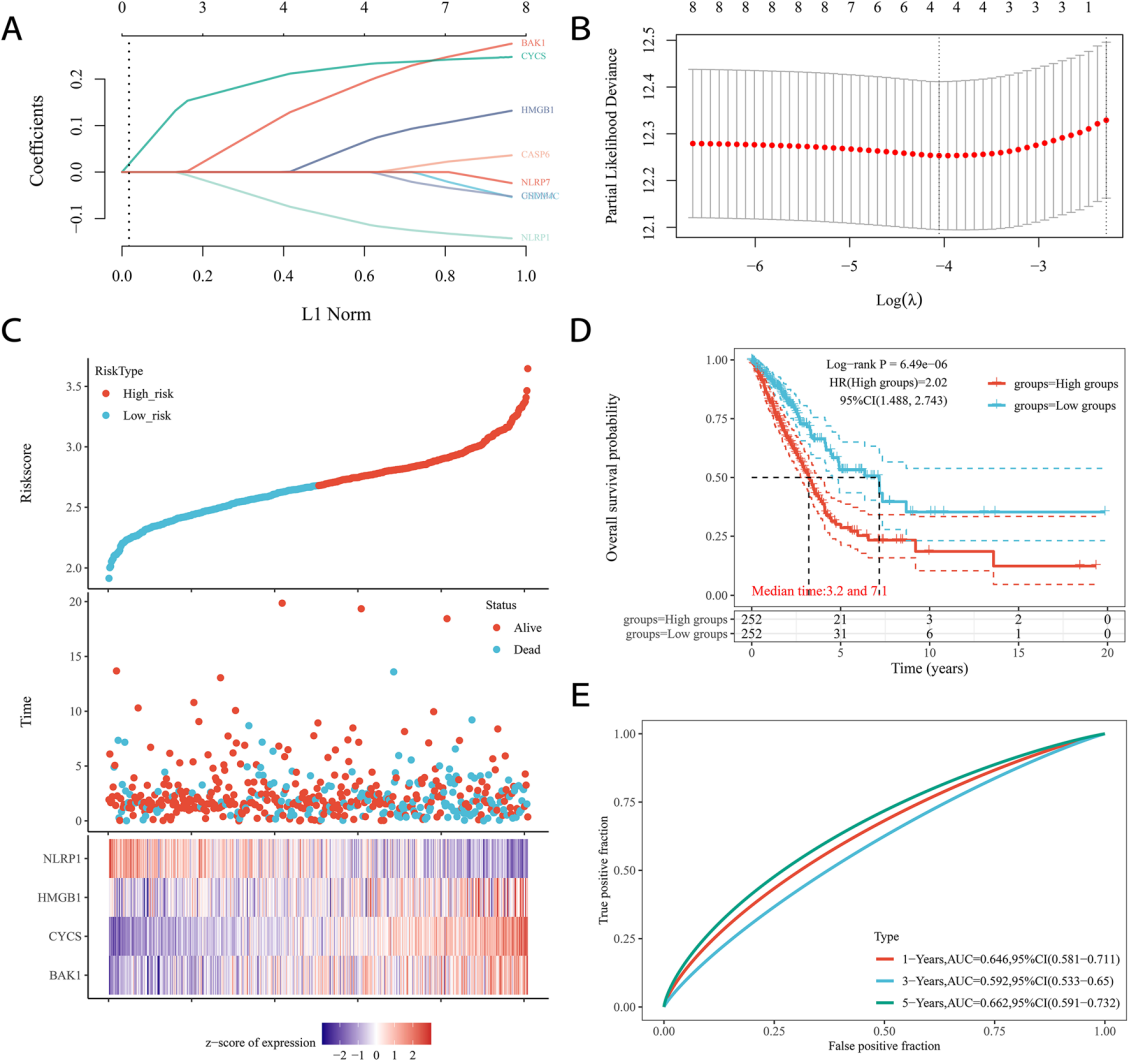
Construction of a prognostic PRGs model. (**A**) LASSO coefficient profiles of the PRGs. (**B**) Plots of ten-fold cross-validation error rates. (**C**) Distribution of risk score, survival status, and the expression of four prognostic PRGs in LUAD. (**D**, **E**). OS curves for LUAD patients in the high- and low-risk group and the ROC curve of measuring the predictive value.

To construct a PRG-based prognostic model, univariate Cox expression analysis was performed to screen the differentially expressed PRGs for prognostic value. We identified 8 genes with significant prognostic value in Kaplan–Meier survival curves (Fig. 4). The results suggested that a poor survival rate in LUAD patients was related to high expression of *BAK1* (Fig. 4A, *p* = 0.014), *CHMP4C* (Fig. 4B, *p* = 0.036), *CYCS* (Fig. 4C, *p* = 0), HMGB1 (Fig. 4D, *p* = 0.004), and CASP6 (Fig. 4E, *p* = 0.034), while a poor survival rate was related to low expression of the other 3 genes: *GSDMA* (Fig. 4F, *p* = 0.047), *NLRP1* (Fig. 4G, *p* = 0.012), and *NLRP7* (Fig. 4H, *p* = 0.02). LASSO Cox regression analysis was performed to construct a prognostic gene model based on the 4 prognostic PRGs (*BAK1*, *CYCS*, *HMGB1*, and *NLRP1*) with the most significant *p*-values (Fig. 5A,B). The risk score = (0.1918) *BAK1 + (0.2309) *CYCS + (0.0635) * *HMGB1* + (− 0.1105) * *NLRP1*. Based on the risk score, LUAD patients were separated into two clusters. The risk score, overall survival time and expression of these four PRGs are presented in Fig. 5C. As the risk score increased, the survival time of patients decreased. *HMGB1*, *CYCS*, and *BAK1* were associated with an increased risk score, while *NLRP1* was a protective gene that was correlated with a decreased risk score (Fig. 5C). Kaplan–Meier curves indicated that patients with a high risk score had a worse overall survival probability than those with a low risk score (median time: 3.2 years in the high-risk subgroup versus 7.1 years in the low-risk subgroup, *p* < 0.001, Fig. 5D). Time-dependent receiver operating characteristic (ROC) curve analysis was applied to evaluate the sensitivity and specificity of the prognostic model, and the results showed that the areas under the ROC curves (AUCs) were 0.646 for 1-year survival, 0.592 for 2-year survival and 0.662 for 5-year survival (Fig. 5E).

### Building a predictive nomogram

**Figure 6:**
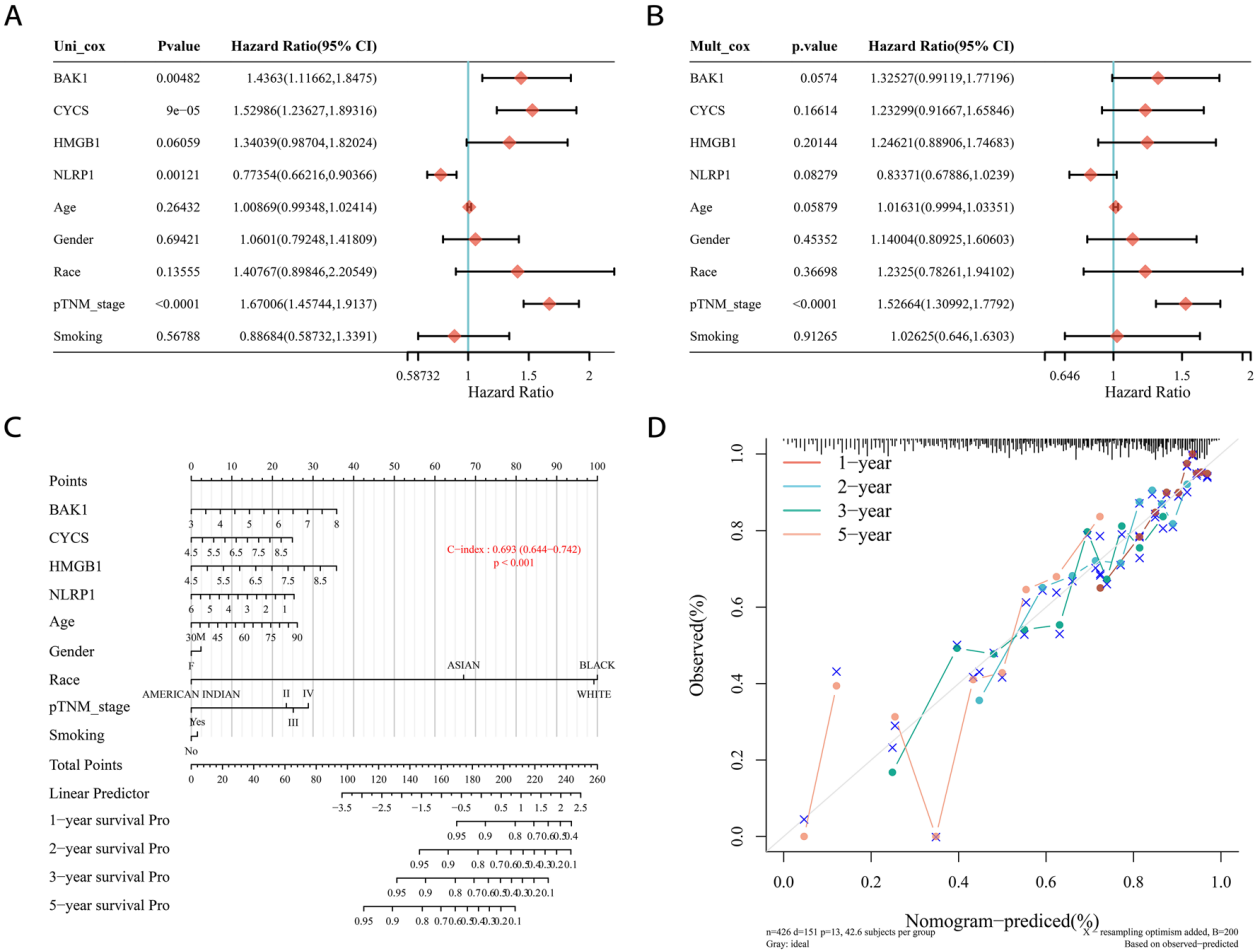
Construction of a predictive nomogram. (**A**, **B**) Hazard ratio and *p*-value of the constituents involved in univariate and multivariate Cox regression considering clinical the parameters and the four prognostic PRGs. (**C**,**D**) Nomogram to predict 1-year, 2-year, 3-year, and 5-year OS ratio of LUSC patients. Calibration curve for the OS nomogram model in the discovery group. A dashed diagonal line represents the ideal nomogram.

To explore the value of these prognostic PRGs for clinical application, we built a nomogram including the clinical features (age, sex, race, pTNM stage, and smoking status) that were generally believed to have a certain impact on the prognosis of LUAD and the 4 prognostic PRGs to predict the survival rate of LUAD patients (Fig. 6). Univariate and multivariate analyses indicated that *BAK1* expression might be a candidate independent factor, similar to pTNM stage, that affects the prognosis of LUAD patients (Fig. 6A,B). A C-index of 0.693 indicated that the nomogram had good predictive value (Fig. 6C). The predictive nomogram suggested that 2-year, 3-year and 5-year overall survival rates could be predicted relatively well according to an ideal model in the entire cohort (Fig. 6D).

### Associations of the tumour mutational burden (TMB) and tumour-infiltrating immune cells with prognostic PRGs

**Figure 7:**
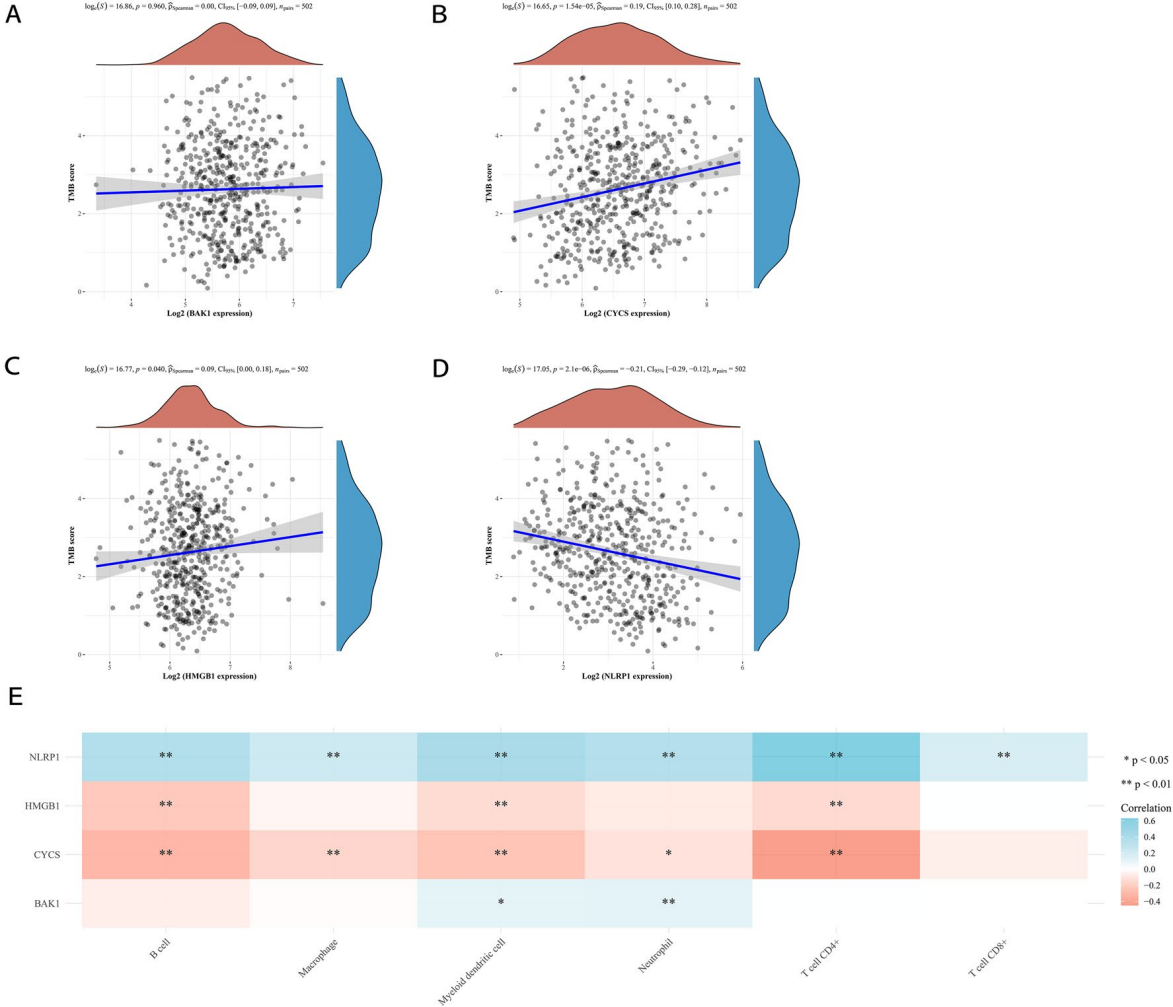
The correlation of prognostic PRGs with TMB and tumour infiltrating immune cells. The correlation of TMB with *BAK1* (**A**), *CYCS* (**B**), *HMGB1* (**C**), and *NLRP1* (**D**) were presented. (**E**) A heatmap of the correlation between prognostic PRGs with tumour-infiltrating immune cells. The horizontal and vertical coordinates represent each prognostic PRG and immune cells, respectively, and the different colours represent correlation coefficients (blue represents positive correlation and red represents negative correlation).

The TMB has been identified as a promising biomarker for predicting immunotherapy responses in patients with NSCLC. To explore the possibility of using prognostic PRGs as biomarkers in immunotherapy for LUAD, we analysed the correlations of prognostic PRGs with the TMB (Fig. 7). The results revealed positive correlations between the TMB and CYCS (Fig. 7B, *p* = 1.54e^−5^) or *HMGB1* (Fig. 7C, *p* = 0.04) and a negative correlation between TMB and *NLRP1* (Fig. 7D, *p* = 2.1e^−6^). However, there was no significant correlation between TMB and BAK1 (Fig. 7A, *p* = 0.96).

Furthermore, to determine the roles of these prognostic PRGs in the tumour immune microenvironment, we performed correlation analysis of tumour-infiltrating immune cells with each prognostic PRG in LUAD by the TIMER database (Fig. 7E). Our results showed positive correlations between *NLRP1* expression and the abundance of B cells (*p* < 0.01), macrophages (*p* < 0.01), myeloid DCs (*p* < 0.01), neutrophils (*p* < 0.01), CD4^+^ T cells (*p* < 0.01) and CD8^+^ T cells (*p* < 0.01). Significant negative correlations were observed between the expression of *HMGB1* and B cells (*p* < 0.01), myeloid DCs (*p* < 0.01), and CD4^+^ T cells (*p* < 0.01). The same negative correlation pattern was also observed for *CYCS* with immune cells, including B cells (*p* < 0.01), myeloid DCs (*p* < 0.01), CD4^+^ T cells (*p* < 0.01), macrophages (*p* < 0.01) and neutrophils (*p* < 0.05). We also found positive correlations between *BAK1* expression and myeloid DCs (*p* < 0.05) or neutrophils (*p* < 0.01).

### Construction of a prognostic model with *BAK1*

**Figure 8:**
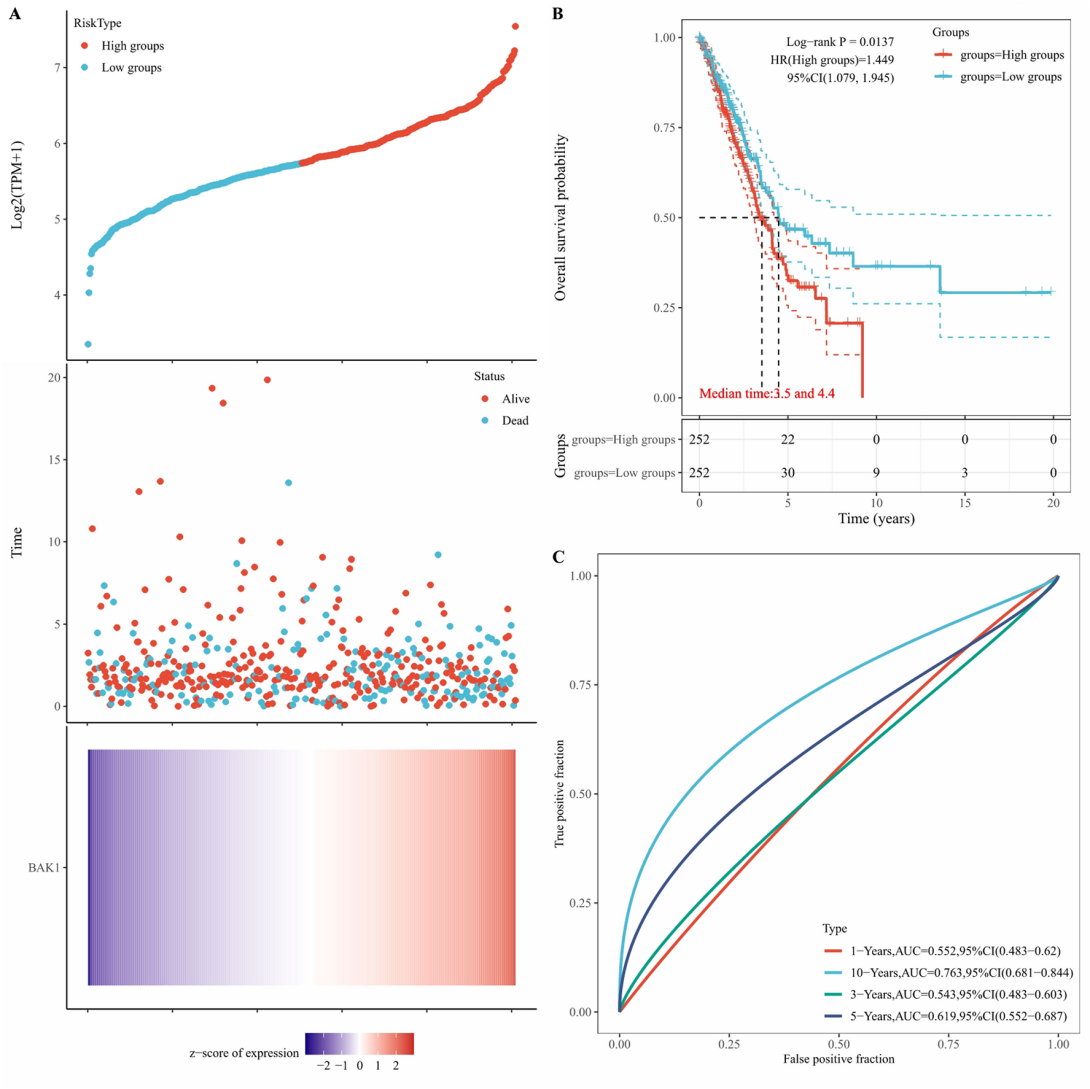
Construction of a prognostic model based on *BAK1* expression. (**A**) Distribution of risk score, survival status, and the expression of *BAK1* in LUAD. (**B**,**C**) OS curves for LUAD patients in the high- and low-risk group and the ROC curve of measuring the predictive value for 1-, 3-, 5-, and 10-year.

As indicated by our analysis of the predictive nomogram results, we concluded that *BAK1* might be an independent prognostic biomarker in LUAD. We further performed prognostic analysis of *BAK1* in patients based on the risk score. The risk score distribution, survival time and *BAK1* expression are shown in Fig. 8A. As the expression of *BAK1* increased, the risk score increased; thus, patients’ risk of death increased accordingly (Fig. 8A). Kaplan–Meier curves revealed that patients with a high risk score presented a worse overall survival probability than those with a low risk score (Fig. 8B; median time: high-risk group, 3.5 versus low-risk group, 4.4; *p* = 0.0137). The AUCs of the risk score were 0.552 for 1 year, 0.543 for 3 years, 0.619 for 5 years and 0.763 for 10 years (Fig. 8C).

### Validation of *BAK1* expression in two independent GEO datasets

**Figure 9:**
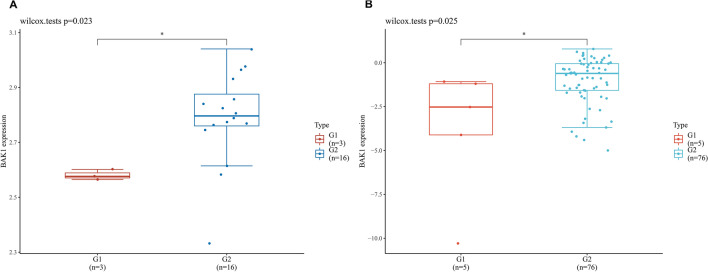
Validation of *BAK1* expression in two independent GEO datasets. (**A**) The expression level of *BAK1* in normal (red) and LUAD (blue) tissues from datasets GSE10799. The horizontal axis represents samples and vertical axis represents gene expression distribution. (**B**) The expression level of *BAK1* in normal (red) and LUAD (blue) tissues from datasets GSE66759. The horizontal axis represents samples and vertical axis represents gene expression distribution.

Two independent GEO cohorts were utilized to validate the *BAK1* expression results in LUAD patients. GSE 10,799 included 3 normal controls and 16 LUAD patients, while GSE 66,759 included 5 controls and 76 LUAD patients. The Wilcox test was used to compare *BAK1* expression between LUAD and normal samples. The results consistently showed that *BAK1* expression was significantly higher in LUAD samples than in control samples (Fig. 9).

## Discussion

Pyroptosis is a newly identified type of programmed cell death that plays a dual role in tumour development and therapeutic mechanisms^7,27^. The process is characterized by rapid rupture of the cell membrane and release of proinflammatory intracellular contents^8,9,13,14^. This unique type of death has led to considerable studies on pyroptosis in various tumours^28–31^. However, the specific role of pyroptosis in LUAD remains unclear.

In this study, we explored 52 currently known PRGs in LUAD and normal tissues and identified that most of them (44/53) were differentially expressed. The three clusters defined based on the differential PRGs did not show any significant differences in overall survival time. However, the abundance of tumour-infiltrating immune cells and immune checkpoint regulators showed significant differences among the three clusters. To further assess the prognostic value of these PRGs, we constructed a 4-gene risk signature via Cox univariate analysis and LASSO Cox regression analysis and found that it had good accuracy for predicting the survival of LUAD patients.

Survival analysis and prognostic models were developed based on four genes (*BAK1*, *CYCS*, *HMGB1*, and *NLRP1*) in this study. Among these, *BAK1* was an independent risk factor for OS in patients with LUAD. High levels of endogenous *BAK* have been observed in both small cell lung cancer and NSCLC cell lines. In addition, increased *BAK* expression was correlated with a poor prognosis in NSCLC patients^32^. Recent research has suggested that *BAK* could be a promising prognostic indicator and potential therapeutic target in lung cancer patients^33^. These studies are in line with our findings, indicating that *BAK1* has a positive correlation with LUAD patient prognosis. We confirmed the high *BAK1* expression level in two independent GEO cohorts. Pyroptosis in colon cancer cell lines can be mediated by *BAK1* or *BAX* alone, and caspase 3 activity is required in *BAK/BAX*-mediated pyroptosis^34^. It is reasonable to hypothesize that abnormal *BAK1* expression could promote the development of LUAD by regulating the pyroptotic pathway^34^. However, the mechanisms by which *BAK1* mediates pyroptosis and leads to LUAD remain elusive. *CYCS*, as a mitochondrial protein that participates in the regulation of cell death, was reported to be an oncogene in LUAD^35^. Previous research has demonstrated that serum *CYCS* levels are correlated with disease progression and a poor prognosis in NSCLC^36^, which is consistent with our current results. Feng et al*.* proved that *HMGB1* was overexpressed in NSCLC tissues^37^. We also found that higher expression of *HMGB1* was correlated with a poor clinical prognosis in LUAD patients. Similar results from Chang et al*.* showed that HMGB/RAGE signalling was significantly associated with patient prognosis, which agrees with our survival analysis results for the TCGA datasets^38^. NLRP1 is recognized as an important component of complexes that can activate caspase-1 directly^39^. Shen et al*.* reported that the *NLRP1* expression in LUAD tissue was considerably lower than that in normal tissues^40^. This decreased *NLRP1* expression was associated with high T and N stages^40^. Consistently, we found that LUAD patients with low *NLRP1* expression had a worse prognosis than those with high expression. Caspase-6 has been shown to be an important regulator in inflammasome activation that could promote the activation of programmed cell death pathways, including pyroptosis, apoptosis and necroptosis (PANoptosis)^41^. Studies have indicated that *NLRP3* inflammasome activation enhances the proliferation and metastasis of the lung adenocarcinoma cell line A549, which are mediated by AKT, ERK1/2, and CREB, and upregulation of SNAIL^42^. Consistent with these findings, in our current study, the expression of *CASP6* was negatively correlated with patient OS.

Recent research has demonstrated that the TMB is a determinant of immune-related survival in a variety of tumours, such as breast cancer and lung cancer^43–46^. In the current study, the prognostic pyroptosis-related genes *CYCS* and *HMGB1* were positively correlated with the TMB, while the expression of *NLRP1* was negatively correlated with the TMB. Pyroptosis was initially found in macrophages but has recently been identified in a variety of immune cells^8,11,47^. Another interesting finding in our results is that the above four prognostic PRGs (*BAK1*, *CYCS*, *HMGB1*, and *NLRP1*) were significantly correlated with immune cell infiltration, which further confirmed that pyroptosis in immune cells might participate in regulating the tumour microenvironment.

Our study has great clinical significance, especially the prognostic model that can be used to predict the prognosis of LUAD patients. Although various advances have been shown to be beneficial to some patients, such as immune checkpoint therapies including programmed cell death protein-1 (PD-1) and T-lymphocyte-associated antigen 4 (CTLA4) blockade, a proportion of patients are resistant to current therapeutic strategies, partly due to the heterogeneity in PD-L1 expression, the TMB and T-cell infiltration in LUAD^48^. Clinically, PRG-based classifiers have the potential to provide a novel approach for identifying novel subtypes of LUAD and personalized treatment for these patients. In terms of immune checkpoint therapy, some of these agents could affect the immune microenvironment through pyroptosis. Therefore, TMB-related PRGs have the potential to be used to guide the curative efficacy of immune checkpoint inhibitors. This study provides new insight into the molecular mechanism underlying LUAD pathogenesis.

Our current study is an exploratory analysis conducted using the TCGA-LUAD cohort, and the results presented here will need to be confirmed in larger datasets. Additionally, the results presented here will need to be confirmed by in vivo and in vitro experiments.

In conclusion, our study identified 44 PRGs that were differentially expressed between LUAD and normal tissues. Based on these PRGs, LUAD patients were classified into 3 subclusters with differential expression levels of tumour-infiltrating immune cells and immune checkpoint regulators. A novel prognostic model based on four PRGs was constructed and used to predict the prognosis of LUAD patients. The correlations of PRGs with the TMB and immune cells may provide evidence that pyroptosis might play an important role in the tumour microenvironment.

## Data Availability

Publicly available datasets were analyzed in this study. This data can be found here: GSE 10,799 (https://www.ncbi.nlm.nih.gov/geo/query/acc.cgi?acc=GSE10799); GSE 66,759 (https://www.ncbi.nlm.nih.gov/geo/query/acc.cgi?acc=GSE66759).

## References

[1] Landman A (2021). 2021 world conference on lung cancer. Lancet Oncol..

[2] Bade BC, Dela Cruz CS, Cancer L (2020). Epidemiology, etiology, and prevention. Clin. Chest Med..

[3] Houssaini MS, Damou M, Ismaili N (2020). Advances in the management of non-small cell lung cancer (NSCLC): A new practice changing data from asco 2020 annual meeting. Cancer Treat. Res. Commun..

[4] Hirsch FR (2017). Lung cancer: Current therapies and new targeted treatments. Lancet.

[5] Succony L, Rassl DM, Barker AP, McCaughan FM, Rintoul RC (2021). Adenocarcinoma spectrum lesions of the lung: Detection, pathology and treatment strategies. Cancer Treat. Rev..

[6] Denisenko TV, Budkevich IN, Zhivotovsky B (2018). Cell death-based treatment of lung adenocarcinoma. Cell Death Dis..

[7] Bertheloot D, Latz E, Franklin BS (2021). Necroptosis, pyroptosis and apoptosis: An intricate game of cell death. Cell. Mol. Immunol..

[8] Hsu SK (2021). Inflammation-related pyroptosis, a novel programmed cell death pathway, and its crosstalk with immune therapy in cancer treatment. Theranostics.

[9] Wu D, Wei C, Li Y, Yang X, Zhou S (2021). Pyroptosis, a new breakthrough in cancer treatment. Front. Oncol..

[10] Fang Y (2020). Pyroptosis: A new frontier in cancer. Biomed. Pharmacother..

[11] Bergsbaken T, Fink SL, Cookson BT (2009). Pyroptosis: Host cell death and inflammation. Nat. Rev. Microbiol..

[12] Lu X, Guo T, Zhang X (2021). Pyroptosis in cancer: Friend or foe?. Cancers (Basel).

[13] Al Mamun A (2021). Role of pyroptosis in cancer and its therapeutic regulation. Eur. J. Pharmacol..

[14] Wang L, Qin X, Liang J, Ge P (2021). Induction of pyroptosis: A promising strategy for cancer treatment. Front. Oncol..

[15] Xia X (2019). The role of pyroptosis in cancer: Pro-cancer or pro-“host”?. Cell Death Dis..

[16] Gao J (2018). Downregulation of GSDMD attenuates tumor proliferation via the intrinsic mitochondrial apoptotic pathway and inhibition of EGFR/Akt signaling and predicts a good prognosis in nonsmall cell lung cancer. Oncol. Rep..

[17] Jiang M, Qi L, Li L, Li Y (2020). The caspase-3/GSDME signal pathway as a switch between apoptosis and pyroptosis in cancer. Cell Death Discov..

[18] Lu H (2018). Molecular targeted therapies elicit concurrent apoptotic and GSDME-dependent pyroptotic tumor cell death. Clin. Cancer Res..

[19] Song J (2021). A novel pyroptosis-related lncRNA signature for prognostic prediction in patients with lung adenocarcinoma. Bioengineered.

[20] Lin W, Chen Y, Wu B, Chen Y, Li Z (2021). Identification of the pyroptosisrelated prognostic gene signature and the associated regulation axis in lung adenocarcinoma. Cell Death Discov..

[21] Liu LP (2021). Identification and validation of the pyroptosis-related molecular subtypes of lung adenocarcinoma by bioinformatics and machine learning. Front. Cell Dev. Biol..

[22] Hartman ML (2020). Non-apoptotic cell death signaling pathways in melanoma. Int. J. Mol. Sci..

[23] Karki R, Kanneganti TD (2019). Diverging inflammasome signals in tumorigenesis and potential targeting. Nat. Rev. Cancer.

[24] Man SM, Kanneganti TD (2015). Regulation of inflammasome activation. Immunol. Rev..

[25] Wang B, Yin Q (2017). AIM2 inflammasome activation and regulation: A structural perspective. J. Struct. Biol..

[26] Zhou Z (2020). Granzyme A from cytotoxic lymphocytes cleaves GSDMB to trigger pyroptosis in target cells. Science.

[27] Zheng Z, Li G (2020). Mechanisms and therapeutic regulation of pyroptosis in inflammatory diseases and cancer. Int. J. Mol. Sci..

[28] Du T (2021). Pyroptosis, metabolism, and tumor immune microenvironment. Clin. Transl. Med..

[29] Ping L, Zhang K, Ou X, Qiu X, Xiao X (2021). A novel pyroptosis-associated long non-coding RNA signature predicts prognosis and tumor immune microenvironment of patients with breast cancer. Front. Cell. Dev. Biol..

[30] Chen X (2021). Turning up the heat on non-immunoreactive tumors: Pyroptosis influences the tumor immune microenvironment in bladder cancer. Oncogene.

[31] Zhang T (2019). Transcription factor p53 Suppresses tumor growth by prompting pyroptosis in non-small-cell lung cancer. Oxid. Med. Cell. Longev..

[32] Park D (2021). Discovery of small molecule bak activator for lung cancer therapy. Theranostics.

[33] Duan J (2021). Identification of a novel autophagy signature for predicting survival in patients with lung adenocarcinoma. PeerJ.

[34] Hu L (2020). Chemotherapy-induced pyroptosis is mediated by BAK/BAX-caspase-3-GSDME pathway and inhibited by 2-bromopalmitate. Cell Death Dis..

[35] Zhang Y, Chen J, Zhao Y, Weng L, Xu Y (2020). Ceramide pathway regulators predict clinical prognostic risk and affect the tumor immune microenvironment in lung adenocarcinoma. Front. Oncol..

[36] Javid J, Mir R, Julka PK, Ray PC, Saxena A (2015). Extracellular cytochrome c as a biomarker for monitoring therapeutic efficacy and prognosis of non-small cell lung cancer patients. Tumour Biol..

[37] Feng A, Tu Z, Yin B (2016). The effect of HMGB1 on the clinicopathological and prognostic features of non-small cell lung cancer. Oncotarget.

[38] Chang YH, Chen CM, Chen HY, Yang PC (2015). Pathway-based gene signatures predicting clinical outcome of lung adenocarcinoma. Sci. Rep..

[39] Kovarova M (2012). NLRP1-dependent pyroptosis leads to acute lung injury and morbidity in mice. J. Immunol..

[40] Shen E (2021). Low expression of NLRP1 is associated with a poor prognosis and immune infiltration in lung adenocarcinoma patients. Aging (Albany NY).

[41] Zheng M, Karki R, Vogel P, Kanneganti TD (2020). Caspase-6 is a key regulator of innate immunity, inflammasome activation, and host defense. Cell.

[42] Hamarsheh S, Zeiser R (2020). NLRP3 inflammasome activation in cancer: A double-edged sword. Front. Immunol..

[43] Hellmann MD (2019). Tumor mutational burden and efficacy of nivolumab monotherapy and in combination with ipilimumab in small-cell lung cancer. Cancer Cell.

[44] Barroso-Sousa R (2020). Prevalence and mutational determinants of high tumor mutation burden in breast cancer. Ann. Oncol..

[45] Xu Q (2021). Multi-omics analysis reveals prognostic value of tumor mutation burden in hepatocellular carcinoma. Cancer Cell. Int..

[46] McGrail DJ (2021). High tumor mutation burden fails to predict immune checkpoint blockade response across all cancer types. Ann. Oncol..

[47] Robinson N (2019). Programmed necrotic cell death of macrophages: Focus on pyroptosis, necroptosis, and parthanatos. Redox Biol..

[48] Topalian SL, Taube JM, Anders RA, Pardoll DM (2016). Mechanism-driven biomarkers to guide immune checkpoint blockade in cancer therapy. Nat. Rev. Cancer.

